# Repercussions of mild diabetes on pregnancy in Wistar rats and on the fetal development

**DOI:** 10.1186/1758-5996-2-26

**Published:** 2010-04-23

**Authors:** Felipe H Saito, Débora C Damasceno, Wilma G Kempinas, Glilciane Morceli, Yuri K Sinzato, Kristin N Taylor, Marilza VC Rudge

**Affiliations:** 1 Faculdade de Medicina de Botucatu, UNESP - Univ Estadual Paulista, Botucatu, Department of Gynecology and Obstetrics, Laboratory of Experimental Research of Gynecology and Obstetrics, São Paulo State, Brazil; 2 Instituto de Biociências de Botucatu, UNESP - Univ Estadual Paulista, Botucatu, Department of Morphology, Laboratório de Biologia e Toxicologia da Reprodução e do Desenvolvimento - ReproTox, São Paulo State, Brazil; 3 Weill Cornell Medical College, New York, USA

## Abstract

**Background:**

Experimental models are necessary to elucidate diabetes pathophysiological mechanisms not yet understood in humans. Objective: To evaluate the repercussions of the mild diabetes, considering two methodologies, on the pregnancy of Wistar rats and on the development of their offspring.

**Methods:**

In the 1st induction, female offspring were distributed into two experimental groups: Group streptozotocin (STZ, n = 67): received the β-cytotoxic agent (100 mg STZ/kg body weight - sc) on the 1st day of the life; and Non-diabetic Group (ND, n = 14): received the vehicle in a similar time period. In the adult life, the animals were mated. After a positive diagnosis of pregnancy (0), female rats from group STZ presenting with lower glycemia than 120 mg/dL received more 20 mg STZ/kg (ip) at day 7 of pregnancy (2nd induction). The female rats with glycemia higher than 120 mg/dL were discarded because they reproduced results already found in the literature. In the mornings of days 0, 7, 14 and 21 of the pregnancy glycemia was determined. At day 21 of pregnancy (at term), the female rats were anesthetized and killed for maternal reproductive performance and fetal development analysis. The data were analyzed using Student-Newman-Keuls, Chi-square and Zero-inflated Poisson (ZIP) Tests (p < 0.05).

**Results:**

STZ rats presented increased rates of pre (STZ = 22.0%; ND = 5.1%) and post-implantation losses (STZ = 26.1%; ND = 5.7%), reduced rates of fetuses with appropriate weight for gestational age (STZ = 66%; ND = 93%) and reduced degree of development (ossification sites).

**Conclusion:**

Mild diabetes led a negative impact on maternal reproductive performance and caused intrauterine growth restriction and impaired fetal development.

## Background

Diabetes mellitus (DM) is characterized by disarrangement of the metabolism of carbohydrates, proteins and lipids caused by the complete or relative insufficiency of insulin secretion and/or insulin action [1]. DM is not a disease but rather a heterogeneous group of syndromes characterized by elevated glucose levels caused by the absolute or relative deficiency of insulin. DM can be classified into: type-1 diabetes (DM1), type-2 diabetes (DM2) and gestational diabetes (DMG). DM1 mainly affects children and young people and results in the destruction of beta (β)-pancreas cells due to autoimmune processes. DM2 refers to the reduction of endogenous insulin in target tissues, particularly liver, muscle and adipose tissue, with relative insulin deficiency. DMG is characterized by the establishment of glucose intolerance, first being identified between 24-26 weeks of pregnancy. DMG may persist after birth and progress to DM2 [1,2].

Pregnancy is characterized by insulin resistance and compensatory hyperinsulinemia and is a period when maternal pancreatic functional reserve is tested. In the absence of pancreatic functional reserve, the pancreas fails to produce insulin levels with relative hypoinsulinism in the second half of pregnancy with consequent maternal hyperglycemia, characterizing DMG [3]. The pathophysiology of DMG is not fully elucidated. Its incidence is variable and occurs in 3 to 8% of pregnant women [4,5].

Impaired reproductive performance is a well-known result of the diabetic syndrome in many mammalian species, including humans. Depending on the severity of the diabetes, pregnancy is frequently impaired. Anovulation, alteration in the estral cycle, reduction in follicular recruitment, insensitivity to exogenous gonadotrophin therapy, steroidogenesis depression and ovarian atrophy are associated with the diabetic state [6,7].

Women with disorders of glucose tolerance present with increased maternal and perinatal morbidity [8], such as spontaneous miscarriages and neonatal morbidity and mortality [9-11]. Improvement in glycemic control during gestation reduces the incidence of spontaneous miscarriages and congenital malformations in the offspring of diabetic women [12].

Epidemiological studies in humans and in experimental animals have shown that offspring of women/rats with gestational/mild diabetes were born macrosomic and developed glucose intolerance during adulthood. Therefore, there is a diabetogenic tendency between different generations in terms of an unfavorable intrauterine environment. This trend was confirmed by experimental tests and studies of human populations, which revealed that inheritance of diabetes is more influenced by maternal than by paternal diabetes [13-15].

Studies in humans that explore the responsible mechanisms for alterations caused by diabetes in pregnancy are limited not only by ethical reasons but also by the multiplicity of uncontrolled variables that may modify the intrauterine environment [16]. Thus, there is a need for appropriate animal models for a better understanding of the diagnosis, pathophysiology and treatment of diabetes.

In order to reproduce the clinical conditions of poorly controlled type-1 diabetes, experimental models are used to produce severe diabetes (glycemia>300 mg/dL) [17-21]. The complications caused in the maternal and fetal organism by severe diabetes are well-known. Additionally, there are models that have been developed to reproduce the clinical conditions of type-2 and gestational diabetes. In laboratory animals, these are classified as mild diabetes (glycemia between 120 and 300 mg/dL). Models of mild diabetes may be induced by administration of different doses of streptozotocin during the neonatal period [22-26] or by injection of streptozotocin during pregnancy [16,27-31]. Sinzato [32] verified that mild diabetes, induced in the neonatal period in female Wistar rats, altered maternal glycemia in the beginning of pregnancy, that caused alterations in the maternal organism and/or in the initial development of the embryo, affecting its implantation and future placental and fetal development. Spada [33], using the same female rats as in the work of Sinzato [32], observed an increase in the activities of glutathione peroxidase in erythrocytes and of catalase in the placenta, demonstrating that the increase of those biomarkers was enough to contain the possible oxidative stress existent after marked hyperglycemia. Kiss [34] observed that female rats with mild diabetes presented with glucose intolerance in the period corresponding to the emergence of gestational diabetes in women. Morceli [35], using a different model of mild diabetes induction (administration of STZ in the neonatal period and in pregnancy), verified that diabetic female rats presented alteration in offspring weight and in the percentage of pre and post-embryonic implantation losses. However, only 48% to 60% of rats with diabetes induced during neonatal or pregnancy period presented with glycemia between 120 and 300 mg/dL, and the results obtained are controversial regarding the presence of fetal macrosomia and placental weight. Additionally, there are no results concerning the effects of mild diabetes on reproductive performance and placental and fetal development. Therefore, the objective of the present study was to evaluate the repercussions of the diabetes, considering two methodologies, on the pregnancy of Wistar rats and on the development of their offspring.

## Methods

### 1. Animals and experimental groups

Newborn Wistar rats obtained from UNESP - Univ Estadual Paulista, São Paulo State, Brazil were used. The rats were maintained in an experimental room under controlled conditions of temperature (22 ± 2°C), humidity (50 ± 10%), and a 12-hour light/dark cycle with *ad libitum *access to commercial diet (Purina rat chow) and tap water. On the day of birth, the rats were randomly distributed into two experimental groups: diabetic (STZ, n = 67) and non-diabetic (ND, n = 14). Procedures and animal handling were performed in accordance with the guidelines provided by the Brazilian College of Animal Experimentation, and authorized by the Ethics Committee for Animal Research of the São Paulo State University (Brazil).

### 2. Experimental Sequence

**Figure 1:**
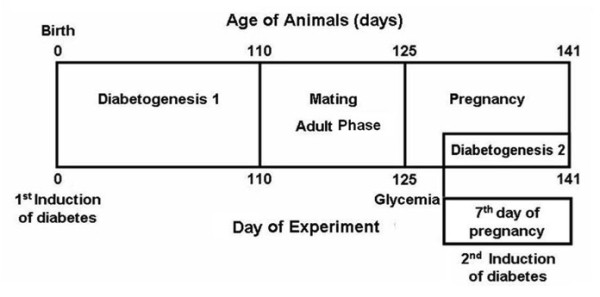
**Experimental sequence**.

The study was divided into four periods: diabetogenesis 1, mating, pregnancy and diabetogenesis 2. The experimental model for diabetes and pregnancy was modified from Calderon and colleagues [36] and the model of induction of mild diabetes was adapted from the methodology described by Portha and colleagues [22] and Tsuji and colleagues [26] (Figure 1).

#### 2.1. Diabetogenesis period 1

Diabetes was induced on the first day of life by subcutaneous administration of streptozotocin (SIGMA Chemical Company, St. Louis, MO), at a dose of 100 mg/kg diluted in 0.1 mol/L of citrate buffer (pH 4.5) [[22,26] modified]. The ND rats received only citrate buffer. Following induction, the rats were kept with their dams with a number of 8 newborns (one newborn/nipple, preferentially females) during the lactation period (21 days). At the end of this period, the dams were euthanized by carbon dioxide inhalation, and the offspring were maintained in the vivarium of the Laboratory of Experimental Research on Gynecology and Obstetrics under controlled conditions.

#### 2.2. Mating period

At adult age (110 days of life), female rats were mated with non-diabetic males. The morning when spermatozoa were found in the vaginal smear was designated gestational day 0. The mating procedure consisted of 15 consecutive days, which comprises approximately three estral cycles in order to obtain the appropriate sample size in each group (n = 15). Non-mated female rats in this period were considered infertile and excluded from the study.

#### 2.3. Pregnancy period

##### 2.3.1. Glycemia

During pregnancy, the females were maintained in individual cages. In the mornings of days 0, 7, 14 and 21, glycemia was determined by a specific glucosemeter (One Touch Ultra - Johnson & Johnson), and the values were expressed in milligrams per deciliter (mg/dL).

##### 2.3.2. Diabetogenesis period 2

Rats from the STZ group presenting with glycemia < 120 mg/dL at day 0 of pregnancy were included in this study and received another dose of streptozotocin (20 mg/kg diluted in 0.1 mol/L of citrate buffer, pH 4.5) at day 7 of pregnancy by intraperitoneal administration route. STZ rats presenting with glycemia ≥120 mg/dL at day 0 of pregnancy were excluded from this study, as because they reproduced results already found in the literature and these animals were used in another study in our lab. ND rats received a similar volume of citrate buffer by the same route of administration.

##### 2.3.3. Maternal reproductive performance

On day 21 of pregnancy, rats were anesthetized with sodium thiopental (Thiopentax - 50 mg/kg) and exsanguinated. Maternal blood was stored in dry and heparinized tubes for biochemical determinations. Laparotomy was then performed to remove the uterine horns for weighing of the litter and subsequent weighing of individual live fetuses and placentas. The ovaries were also removed and the corpora lutea counted and analyzed under a stereomicroscope. The numbers of implantations, resorptions (embryonic deaths), live and dead fetuses were counted as well the rate of pre-implantation loss was calculated by the following formula: number of corpora lutea - number of implantation sites × 100/number of corpora lutea. The rate of post-implantation loss was calculated by the following formula: number of implantations - number of live fetuses × 100/number of implantations [37]. In the absence of visible embryonic or fetal development, the uterus was placed in reactive Salewski [38].

##### 2.3.4. Insulin determination

For measurement of insulin concentration, blood samples collected in heparinized tubes were centrifuged at 2500 × g and 4°C for 10 minutes to obtain plasma and stored in a freezer at -80°C. Blood from 15 rats (n = 7 for the ND group, n = 8 for STZ group) was drawn to perform the measurement by ELISA following the protocol of the manufacturer (Mercodia - Rat Insulin).

##### 2.3.5. Fetal classification

Term fetuses were removed and weighed. The fetuses were classified by the mean ± 1.7 SD according to the mean values of fetal weights of the non-diabetic group (ND): as small for pregnancy age (SPA) when weight was smaller than ND mean - 1.7 SD; appropriate for pregnancy age (APA) when weight was included in ND mean ± 1.7 SD; and large for pregnancy age (LPA) when weight was greater than ND mean + 1.7 SD [31].

#### 2.4. Analysis of fetal anomalies

The fetuses were weighed and analyzed for the presence of external anomalies. After this analysis, half of the fetuses were fixed in Bodian's solution and serial sections were prepared as described by Wilson [39] for visceral examination. The remaining fetuses were prepared for skeletal examination by the staining procedure of Staples & Schnell [40]. Besides the skeletal analyses, the counting of the ossification sites was performed according to methodology proposed by Aliverti [41], which determines the degree of fetal development.

### 3. Statistical analysis

The analysis of variance (ANOVA) was performed to evaluate the main effects and interactions. For the purposes of testing, the normality of the data was verified and, when necessary, transformation was used. In the case of significant difference, Student-Newman-Keuls test was performed. For the classification of fetal weights (SPA, APA, LPA), the Chi Square Test was performed to compare distributions. For external and internal fetal anomalies, Zero-inflated Poisson (ZIP) Test was applied. A p-value of less than 0.05 was considered significant.

## Results

### 1. Glycemia

**Figure 2:**
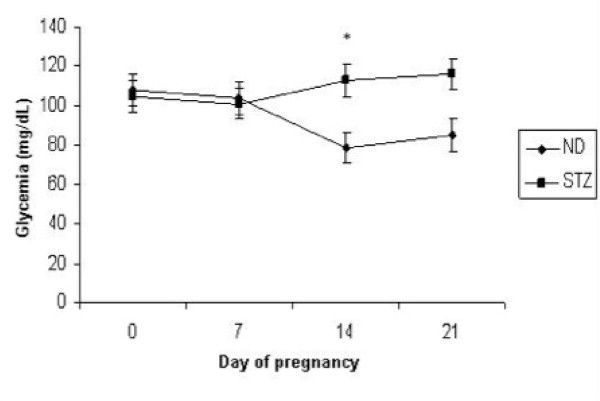
**Glycemia of dams throughout the pregnancy**. Glycemia were taken on days 0, 7, 14 and 21 of pregnancy from mild diabetic (STZ) and non-diabetic (ND) rats. Data are reported as mean ± standard deviation. *p < 0.05 - Student-Newman-Keuls Test.

Mean blood glucose concentration of rats during pregnancy is shown in Figure 2. In the beginning (day 0), at 7 and 21 days of pregnancy, maternal glucose levels did not differ between experimental groups (p > 0.05). On the 14th day of pregnancy, there was an increase in mean glucose in the STZ group.

### 2. Maternal reproductive performance and insulin determination

There were significantly decrease of full-term pregnant rats in STZ group compared to the ND group. The STZ group showed no significant difference in the mean number of live fetuses and fetal weight compared to the ND group. Regarding maternal weight gain, number of corpora lutea and implantations and percentage of losses pre and post-implantation, the STZ group showed a decreased incidence (p < 0.05) compared with ND group (Table 1). At the end of pregnancy, the STZ group showed no significant difference (p > 0.05) in insulin levels when compared with ND group (Table 1).

**Table 1:** Reproductive performance and maternal insulin from mild diabetic (STZ) and non-diabetic (ND) rats.

Group	ND	STZ
Number of used rats	15	67
Number of pregnant rats at term	15	28
Number of corpora lutea	14.00 ± 1.60	10.30 ± 2.00*
Number of implantations	13.23 ± 1.30	7.31 ± 3.10*
Number of live fetuses	161	108
Pre-implantation loss (%)	5.14	22.00*
Post-implantation loss (%)	5.77	26.10*
Fetal weight (g)	5.27 ± 0.28	4.94 ± 0.62
Maternal insulin (μg/L)	0.56 ± 0.51	0.78 ± 0.62

Data are reported as mean ± standard deviation

*p < 0.05 - Student-Newman-Keuls Test

### 3. Classification of fetal weights

**Figure 3:**
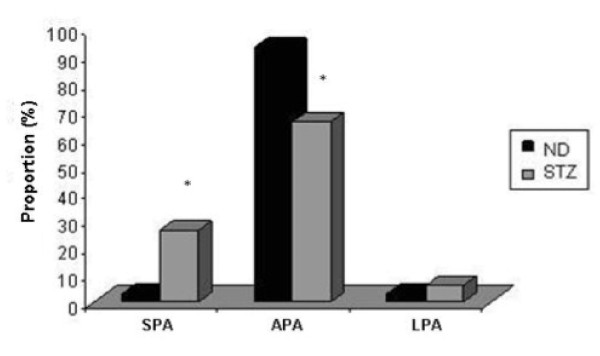
**Fetal weight classification of offspring born to mild diabetic (STZ) and non-diabetic (ND) rats**. Fetal weight classification is defined as small for pregnancy age (SPA), appropriate for pregnancy age (APA) or large for pregnancy age (LPA). *p < 0.05 -Chi Square Test.

The fetuses of rats in the ND group had appropriate weights for age of pregnancy (APA - 93%). The remaining fetuses were evenly distributed between small for pregnancy age (SPA - 3%) and large for pregnancy age (LPA - 3%). The STZ group, 66% of fetuses were classified as APA, 26% as SPA and 8% as LPA. There was a significant increase in SPA fetuses and reduction of APA fetuses (p < 0.05) in STZ group compared to ND group (Figure 3).

### 4. Analysis of fetal anomalies

#### 4.1. Ossification sites

The fetuses from STZ rats showed a significant difference in number of anterior and posterior phalanges, metatarsus and total ossification sites (p < 0.05) compared with fetuses from ND rats (Table 2).

**Table 2:** Ossification sites of fetuses from mild diabetic (STZ) and non-diabetic (ND) rats.

Ossification Sites	ND	STZ
Anterior phalange	3.99 ± 0.04	2.65 ± 1.20*
Metacarpus	4.00 ± 0.00	3.86 ± 0.31
Posterior phalange	2.58 ± 1.09	0.78 ± 1.60*
Metatarsus	4.98 ± 0.05	4.35 ± 0.43*
Caudal vertebrae	4.39 ± 0.81	4.24 ± 1.42
Sternebrae	6.00 ± 0.00	5.95 ± 0.12

**Total**	25.93 ± 1.50	21.82 ± 4.39*

Data are reported as mean ± standard deviation

*p < 0.05 - Student-Newman-Keuls Test

#### 4.2. Skeletal and visceral examination

There were not skeletal and visceral anomalies in the fetuses from rats of the two experimental groups presented with statistical differences (p > 0.05) (Table 3 and 4, respectively).

**Table 3:** Frequency of skeletal anomaly of fetuses from mild diabetic (STZ) and non- diabetic (ND)rats.

Skeletal Anomaly (%)	ND (n = 81 fetuses)	STZ (n= 41 fetuses)
Number of fetal skeletal anomalies/dams	10/15	17/11
Abnormally shaped sternebrae	2/81 (2.47)	9/41 (21.95)
Absent sternebrae	0/81 (0.00)	1/41 (2.43)
Incomplete ossification of sternebrae	0/81 (0.00)	3/41 (7.32)
Bipartite cervical nuclei	3/81 (3.70)	0/41 (0.00)
Incomplete ossification of cervical nuclei	5/81 (6.17)	0/41 (0.00)
Non-ossified sacral vertebrae	0/81 (0.00)	1/41 (2.43)
Non-ossified caudal vertebrae	0/81 (0.00)	3/41 (7.32)

p > 0.05 - non-significant

**Table 4:** Frequency of visceral anomaly of fetuses from mild diabetic (STZ) and non-diabetic (ND) rats.

Visceral Anomaly (%)	ND (n = 74 fetuses)	STZ (n = 37 fetuses)
Number of fetal visceral anomalies/dams	86/15	27/12
Distended bladder	1/74 (1.35)	2/37 (5.40)
Dilated renal papilla	0/74 (0.00)	4/37 (10.81)
Absent renal papilla	0/74 (0.00)	1/37 (2.70)
Small renal papilla	0/74 (0.00)	1/37(2.70)
Ectopic kidney	36/74 (48.65)	4/37 (10.81)
Hydroureter	19/74 (25.70)	5/37 (13.51)
Hydronephrosis	1/74 (1.35)	0/37 (0.00)
Crystalline altered	0/74 (0.00)	1/37 (2.70)
Enlarged nasal cavity	9/74 (12.16)	0/37 (0.00)
Abnormal palate	2/74 (2.70)	1/37 (2.70)
Dilated trachea	5/74 (6.76)	4/37 (10.81)
Dilated esophagus	1/74 (1.35)	3/37 (8.11)
Dilated pulmonary trunk	9/74 (12.16)	0/37 (0.00)
Dilated cerebral ventricle	2/74 (2.70)	1/37 (2.70)
Small testis	1/74 (1.35)	0/37 (0.00)

p > 0.05 - non-significant

## Discussion

The mild diabetes model in pregnant female rats using induction with STZ at two different timepoints led to increased glycemia at day 14 of pregnancy. This discrete increase of glycemia at day 14 of pregnancy is compatible to the increase found in the DMG in women. The elevation of maternal glycemia in the third week of pregnancy may correspond clinically to the 24th and 26th weeks of gestation in women when the diagnosis of DMG, characterized by glucose intolerance is made [1,2]. This finding evidences that the female rats of the STZ group developed, during the pregnancy period, diabetes that simulates human DMG.

It was observed that the plasma insulin concentrations between the two groups were similar at the end of the pregnancy, corroborating with results found by Triadou and colleagues [24].

In the present study, the female rats of the STZ group presented with a reduction in the number of corpora lutea, evidencing a reduction in the number of oocytes liberated during the ovulation process. Similarly, there fewer fertilized mature oocytes and implanted embryos as a function of the altered intrauterine environment in those female rats. Due to the alterations in the number of corpora lutea and implantations in the female rats of the STZ group there was a significant increase in the rates of pre and post-implantation losses, respectively. However, the embryos that were implanted did develop, as there was not an alteration in the number of live fetuses.

In uncontrolled human diabetes, there is evidence of an increased rate of intrauterine deaths and abortions, and in rats with severe diabetes, there is an increase in embryonic deaths [17,42-44]. DMG is associated with increased morbidity (hypoglycemia, hypocalcemia, polycythemia, hyperbilirubinemia, and macrosomia) and fetal mortality [45], and the pregnant diabetic is more prone to exhibit an increased incidence of miscarriage compared to non-diabetic pregnant women [46].

The rats that received the β-cytotoxic agent in the neonatal period and at day 7 of pregnancy began the gestational period with reduced weight because of metabolic changes caused since the induction of diabetes in the neonatal period. Besides, our results showed that rats of the STZ group showed no difference in mean fetal weight. However, the analysis of the classification of the weights of fetuses from STZ rats showed increased rates of fetuses classified as small for pregnancy age (SPA) and reduced rates of fetuses classified as appropriate for gestational age (APA), even dams with glucose not exceeding 300 mg/dL (severe diabetes). Given that there was another application of streptozotocin at day 7 of pregnancy and this is the organogenesis period, the hypoglycemia may have altered the growth and development of fetuses from STZ rats. Furthermore, the method of administration of diabetogenic drugs during pregnancy in rats may have also interfere with the viability of the fetuses, as the route of administration was intraperitoneal.

STZ rats presented SPA fetuses. These data are not consistent with the literature data, which shows that maternal hyperglycemia less than 300 mg/dL causes fetal hyperglycemia, stimulating pancreatic beta cells to secrete more insulin, leading to excessive growth of the fetus or macrosomia [15,47]. The intrauterine growth restriction (fetuses classified as SPA) may also be explained by maternal hyperglycemia above 300 mg/dL (severe diabetes), which leads to depletion of functional fetal pancreas with reduced insulin production, causing an insult to fetal growth [15,48], and thus a higher proportion of fetuses SPA. However, this explanation is not valid for the type of induction used in our study, as the rats did not show at any time of pregnancy the high blood glucose levels that characterized severe diabetes.

Fetal growth is a complex process that depends on the genotype and epigenotype of the fetus, maternal nutrition, the availability of nutrients and oxygen to the fetus, intrauterine insults, and a variety of growth factor and proteins of maternal, fetal and placental origin [49]. During the first trimester of pregnancy, embryonic growth might be controlled at the level of the individual organs by nutrient supply and by locally active growth factors. Later, fetal growth depends essentially upon maternal-placental cooperation in delivering nutrients to the fetus. Therefore the major hole of hormones in fetal growth is to mediate utilization of available substrate. Fetal growth seems to be regulated by fetal insulin, insulin-like growth factor (IGF-1) and IGF-2, while growth hormone (GH) has only a secondary hole to play [50]. During pregnancy, placental growth hormone (PGH) is the prime regulator of maternal serum IGF-1. The alterations in the maternal GH/IGF axis may lead to permanent pathological fetal programming of the IGF axis [50], causing late consequences of poor fetal environment reflected in intrauterine growth restriction, as confirmed by our results.

Although the fetuses of ND rats have shown high frequencies of abnormalities, in visceral analysis, in the comparison between the two groups these values did not differ significantly. The enlarged ureter is an alteration easily recognized by the format in "S", it can be associated or not to the congenital hydronephrosis or any pelvic alterations and, usually, it is transitory in rodents [51]. However, the high rate of ectopic kidney in control rats is unclear. The literature evidences that 10 to 20% of the newly born of control female rats (non-diabetic) present that type of smaller visceral malformation. This result seems not to be exclusive of the diabetes [52-54] and does not exist definition about the critical period of the pregnancy of female rats for the development of these structures. Therefore, the mild diabetes induced in the neonatal period and during pregnancy showed no teratogenic effect on fetuses.

## Conclusion

The mild diabetes led a negative impact on maternal reproductive performance, as evidenced by the increase in the percentages of embryofetal loss and changes in embryo-fetal development confirmed by the presence of fetuses small for gestational age of pregnancy (intrauterine growth restriction). Our study also characterized the presence of a smaller number of rats with hyperglycemia in pregnancy to term and their descendants did not have macrosomia, but showed retardation of fetal development as confirmed by decreased number of ossification sites.

## Abreviations

DM: diabetes mellitus; DM1: type-1 diabetes; DM2: type-2 diabetes; DMG: gestational diabetes; SPA: small for pregnancy age; APA: appropriate for pregnancy age; LPA: large for pregnancy age.

## Competing interests

The authors declare that they have no competing interests.

## Authors' contributions

FHS participated in the acquisition, analysis and interpretation of data and helped to draft the manuscript. DCD conceived the study, participated in its design, coordination, analysis and interpretation of data and helped to draft the manuscript. WGK helped to draft the manuscript. GM and YKS participated in the acquisition, analysis and interpretation of data and helped to draft the manuscript. KNT and MVCR helped to draft the manuscript. All authors read and approved the final manuscript.
